# Low-frequency electromagnetic fields as an alternative to sanitize water of drinking systems in poultry production?

**DOI:** 10.1371/journal.pone.0220302

**Published:** 2019-07-25

**Authors:** Rafael H. Mateus-Vargas, Nicole Kemper, Nina Volkmann, Manfred Kietzmann, Jessica Meissner, Jochen Schulz

**Affiliations:** 1 Institute for Animal Hygiene, Animal Welfare and Farm Animal Behavior, University of Veterinary Medicine Hannover, Foundation, Bischofsholer Damm, Hannover, Germany; 2 Department of Pharmacology, Toxicology and Pharmacy, University of Veterinary Medicine Hannover, Foundation, Buenteweg, Hannover, Germany; University of Hong Kong, HONG KONG

## Abstract

Low-frequency electromagnetic fields (LF-EMF) may present an alternative to conventional sanitation methods of water supply lines in animal production. The objective of this study was to evaluate the effect of the application of LF-EMF on bacterial concentrations and biofilms at scale-models of different drinking systems (circulating and non-circulating) conventionally used in poultry holdings. Treated systems were equipped with commercial devices producing pulsed electromagnetic signals of low frequency up to 10,000 Hz; max. 21 mT. Exposure of water to LF-EMF resulted in changes of the culturable bacterial counts, although with high standard deviations. Differing between systems types, LF-EMF treatment seemed to be responsible either for a limitation or for an increase of colony forming unit counts, with partly statistically significant differences, especially in early stages of treatment. In contrast, neither biofilm formation nor counts of cells suspended in water differed between treated and control lines over 28 days of experiment, as determined by fluorescence microscopy. Although this study indicates that LF-EMF may influence culturability of water microorganisms, no clear inhibitory effects on bacterial biofilm formation or on planktonic microbes by LF-EMF treatment were confirmed in the experiments.

## Introduction

Assurance of a good microbiological quality of drinking water plays an important role in animal production, as it direct contributes to animal health and consequently to optimal production performance [1, 2]. In poultry operations, drinking water lines are especially vulnerable for microbial contamination [3]. After reaching water, pathogens distribute throughout the water systems, colonize biofilms, and pose an important infection risk to individuals consuming water [4]. Since biofilms act as important environmental reservoirs for pathogens, once they have reached and developed in existing biofilms [5], one remarkable challenge to assure the microbiological quality of drinking water may be the effective control of biofilm formation in water distribution systems [6].

Water line sanitation is based on the use of physical cleaning, mostly by flush washing, and/or by the application of different chemical disinfectant compounds alone or in combination, including among others: acidifiers, chlorine or hydrogen peroxide [7]. Sanitation processes, which were individually adapted to the respective conditions of each poultry operation, are an essential part of an effective biosecurity program [8]. Despite the variety of compounds and protocols available, conventional sanitation has a limited effect on control of biofilm formation, as effectivity depends on different factors such as concentration of chemical compounds in water lines, exposure time, composition of biofilm and pipe line material [9–12]. In the last years, the results of different experimental studies have suggested the use of low frequency electromagnetic fields (LF-EMF) as an alternative to conventional sanitation methods for the control of biofouling. For example, LF-EMF treatment using frequencies above 1 kHz has been reported not just to affect mineral scale of pipe lines, but also to reduce microbial mass attached on inner surfaces of water distribution networks [13–17]. In fact, even the use of frequencies below 300 Hz may modify the adhesion ability [18, 19], growth rate [20], viability [21], as well as antibiotic susceptibility of microorganisms [22]. Effects on particle aggregation are suspected to be related with alterations of physical interaction forces of particle surfaces, including those at cellular membranes [14, 15]. Intracellularly, for instance, a reorganization of water [23], free radical formation [24], as well as changes in the molecular structure of DNA [25] have been suggested to be caused by EMF treatment. Despite the current knowledge on its repercussions under laboratorial conditions, the biological effects on microbes in drinking water are still not well understood [26]. Since a continual assurance of water quality poses an important challenge in animal husbandry [8], it is of interest to study the applicability of novel sanitation technologies in different stages of production. Although LF-EMF has been used in poultry farms to control lime scale formation in water supply lines [14], to the best of our knowledge, there is no published data about the effects of this technology on the hygienic status of water distribution lines in animal husbandry.

The objective of this study was to investigate the sanitary effects of the application of a commercial available LF-EMF device (22 mT; 350 to 10,000 Hz) by determining bacterial loads in water and biofilm formation in models of drinking systems commercially available for poultry holdings over 28 days.

## Material and methods

### Experimental system

**Fig 1:**
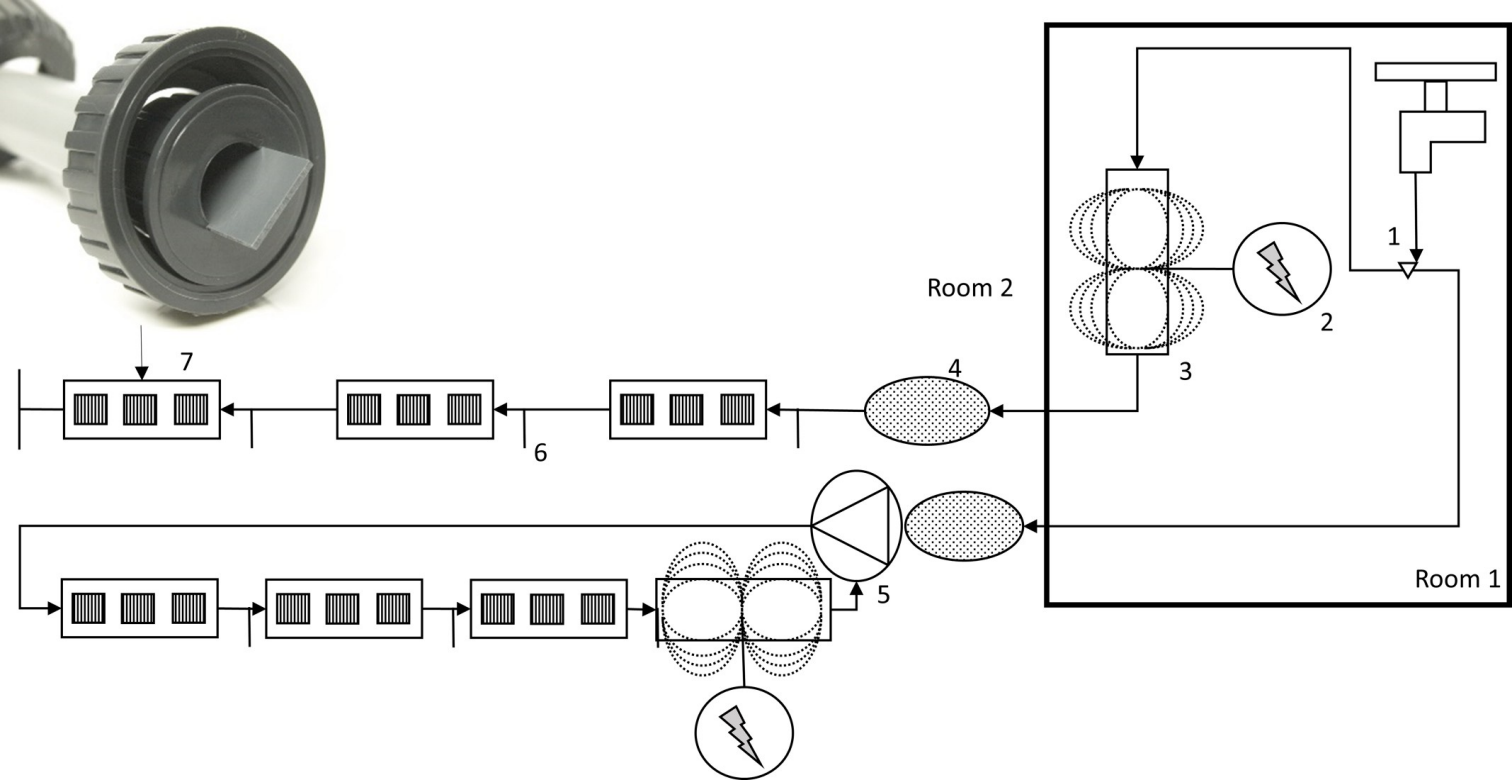
Schematic representation of models of drinking systems. Water supply (1); control unit (2); treatment unit (3); pressure regulator (4); peristaltic pump (5); sampling nipple (6); replaceable PVC-pipes with PVC-coupons (7). Picture shows position of coupons in replaceable pipes. Bold frame indicates a separate room. Untreated systems (controls without the electromagnetic device) are not shown.

Models of commercial drinking systems for poultry (polyvinyl chloride, PVC) were assembled in parallel for the realization of the experiments. The systems consisted of two non-circulating (21.2 m; single-piped) and two circulating (22.8 m; closed loop configuration) nipple pipelines with a total water capacity of 10.3 l and 14.7 l each, respectively (Lubing, Barnstorf, Germany). Controls were installed at a distance of 1.35 m from treated lines. Each drinking system comprised a pressure regulator unit, nipple pipes, cups and breather units. Pressure in the drinking systems was set to approximately 15 mbar at the beginning of the experiments. Circulating nipple pipes were further equipped with a circulation unit which produced a flow rate of 0.7 l min^-1^. Whilst water in circulating pipes was in constant movement, the water in single piped systems only flowed through pipe lines when water pressure dropped in the system, e.g. when sampling was performed. In order to assess the effects of LF-EMF treatment of water, one model of each drinking system type (treated) was further equipped with a commercial electromagnetic device (PJ-25I HST for non-circulating and AJM-20I HST for circulating pipelines, Bauer Watertechnology Ltd. Vantaa, Finland). Devices comprised a control unit and a treatment unit. Electromagnetic signals were generated in the control unit and transported through a cable to the treatment unit, in which the water was exposed. Control unit is furthermore equipped with sensors and warning lights, which alarm when no signal is being generated. For the conduction of the experiments, two treatment methods, which may be more likely to be used at farm level, were selected: For the circulating system, the device was placed directly in the nipple pipe resulting in a continuous circulation of water through the device. In contrast, the electromagnetic device was positioned at the water supply. Consequently, water of the treated single-piped system passed only once through the device, namely before reaching the drinking system. The devices generated an altering frequency magnetic field (350 to 10,000 Hz) with a maximum amplitude of 22 mT orthogonal to the water flow. Architecture of the treatment unit as well as the distance drinking systems prevented an influence of the signals generated for the treated lines on controls. For the study of biofilm formation, drinking systems were further equipped with replaceable 18 cm-pieces of PVC-pipes at three positions along the pipe lines. Every replaceable pipe was provided with PVC-coupons (3 cm long and 2 cm wide). A schematic representation of the pilot-models is shown in Fig 1. Images of the models are included in the supporting information (S1 Fig).

### Test procedure

The water used in the experiments was coming directly from the water provided by the Hanover water supplier. Water is produced mostly from groundwater and has to fulfill all requirements of the German Drinking Water Ordinance [27]. Physical-chemical characteristics of water were assessed at the beginning of the experiments with samples taken directly at place of water supply (S1 Table). Before every trial, drinking systems were cleaned and disinfected through the alternate use of commercial products. After manufacturer recommendations for application in poultry farms, pipelines were first filled with water containing 5% of DESINTEC AH-tec for 1 h (VitaVis GmbH, Münster, Germany). After flushing the pipelines, water that contained 1% of Virkon H2O was allowed to sit for 1 h in water lines (Antec International Limited, Suffolk, United Kingdom). Finally, water lines were thoroughly flushed again, electromagnetic devices of treated pipelines were switched on, and all drinking systems were filled with drinking water. Treated and control systems were monitored simultaneously during 28 days. Functionality of the devices was monitored during the experiments weekly by controlling the warning lights. Experiments were conducted in two independent trials under identical conditions in terms of water exposure to LF-EMF in the period between June and October 2017. Drinking habits of the animals were not simulated in this study and thus, water was only extracted of the systems during sampling.

### Sampling

Both water and biofilm samples were taken at three sampling points alongside the drinking systems (Fig 1). To sample water, one nipple per sampling point was first drowned in alcohol. After 3 min, nipples were allowed to dry and initial 100 ml of water were aseptically taken directly of the disinfected nipples and discarded. Subsequently, 100 ml and 20 ml of water were sampled in sterile clear glass laboratory bottles from each nipple for microbiological analyses as well as for pH and temperature measurements, respectively. Temperature and pH were determined direct after sampling of each nipple. Temperature and pH were measured with PHenomenal (pH portable set pH 1100H, VWR International bvba, Leuven, Belgium).

Following water sampling, two coupons were taken out of the pipes at each sampling point to analyze biofilms. Directly after removal, coupons were rinsed three times each with 10 ml sterile NaCl 0.9% to wash non-adherent cells. One of these coupons was then immersed in a 50 ml centrifuge tube containing 20 ml NaCl 0.9%. The other coupon was placed in a separate centrifuge tube with 20 ml 0.01% PBS-Tween 20. Obtained coupons were intended for fluorescence microscopic and microbiological analyses, respectively. Both, water samples and coupons were stored by 4°C until analysis, which took place within the next four hours after sampling. The effect of LF-EMF on microbial load and biofilm growth on coupons was analyzed weekly by plate counting and fluorescence microscopy.

### Microbiological analysis

For microbiological examinations, centrifuge tubes containing PVC-coupons and 0.01% PBS-Tween 20 were first sonicated in a Sonorex Super 10 P (RK 1028 P, Bandelin electronic GmbH & Co., Berlin, Germany) with a frequency of 35 kHz and a power output of 1,000 W for 10 min. Following sonication, both sides of each slide were thoroughly scraped off using a sterile cell scraper perpendicular to the surface. Subsequently, surfaces of the coupon were thoroughly wiped with a sterile swab. Biofilm material together with PVC-coupon, cell scrapers head and swab were collected in the PBS-Tween 20 solution and vortexed for 1 min for homogenization. This combined approach was chosen because of detachment efficiency of biofilm, recovery rate of cells in supernatant, as well as repeatability observed in preliminary trials (not shown). Due to the close similarity of conditions inside the pipe lines and taking into account labor´s capacity, sample pooling seemed to be a feasible option to obtain a general overall view of the bacterial community along the drinking systems during the experiments [28]. Hence, water samples, taken at the three sampling points of each water line, were first pooled and mixed thoroughly by vortexing for 3 min after arrival at the laboratory.

Pooled water samples and biofilm material were analyzed via the heterotrophic bacteria count and the number of pseudomonads. Briefly, heterotrophic bacteria were assessed by plating serial dilutions in triplicate using blood-agar base (Carl Roth GmbH & Co. KG, Karlsruhe, Germany). Pour-plate and surface plating technique were performed for water and biofilm samples, respectively. Inoculated plates were incubated either at 22°C or at 36°C for 48 h. For determination of the heterotrophic plate count (HPC; HPC-22 or HPC-36), all colonies were counted and weighted average was calculated to determine the number of colony forming units (cfu) per ml for water samples. Biofilm results were further normalized to cfu per surface area (cfu cm^-2^). Similarly, for the assessment of pseudomonads, aliquots of homogenized dilutions were spread on Pseudomonas CFC Agar-plates (Oxoid GmbH, Wesel, Germany). Following 24 h incubation at 30°C, all presumptive colonies were counted to determine the cfu of pseudomonads in water and biofilm samples.

### Fluorescence microscopy

To visualize quantitative effects on microbial cells to LF-EMF in the systems, both planktonic in water samples and sessile cells on PVC-coupons were stained with DNA-specific stains and analyzed under epifluorescence microscopy. Total counts of cells in serial diluted water samples were determined through use of DNA binding stains SYTO 9 (Life Technologies Corporation, Eugene, USA) and propidium iodide (PI; AppliChem GmbH, Darmstadt, Germany) using a protocol adapted specially for this study. The protocol was based on data published by Boulos et al. [29]. Briefly, 20 μl of Mowiol 4–88 (Carl Roth GmbH & Co. KG, Karlsruhe, Germany), as well as SYTO 9 at a final concentration of 0.25 μM were at first added to a 10 ml homogenized sample. After 25 min incubation in the dark at 22°C, samples were counterstained with PI adding 5 μl of stock solution (4 mg ml^-1^) and incubated for additional 5 min. After incubation, water samples were thorough homogenized and consecutively filtered through 0.2 μm Isopore black polycarbonate membranes (Merck KGaA, Darmstadt, Germany). Polycarbonate filters were then air dried, mounted on a glass slide and covered with a glass cover slip. Stained bacteria were determined using an Axio Imager M2 fluorescence microscope coupled with an Apotome (Carl Zeiss, Jena, Germany) under 450–490 nm and 540–580 nm filter for excitation of SYTO 9 and PI stained cells, respectively. Emissions were observed at 528 nm (SYTO9) and 645 nm (PI). This staining combination is widely used for the enumeration of bacteria and allows the assessment of the total number of cells (SYTO9; green) and the fraction of dead cells (PI; red), as PI only penetrates bacteria with damaged membranes [29]. One dilution step showing an average of ≤150 cells in fields was chosen per sample for further quantification. The number of cell and cell aggregates was estimated from counts of 22 microscopic fields (at 100x/ 1.4 oil immersion objective) randomly selected. For further analyses, each cell aggregate was counted as a cell unit, since it was often not possible to differentiate single cells despite previous thorough homogenization of water samples. Sample pictures of microscopic fields of filters with cell and cell aggregates of water samples were included in the supporting information (S2 Fig).

The microbial colonization was determined on PVC-coupons, which were previously immersed in 20 ml NaCl 0.9% using 4,6-di-amidino-2-phenylindole (DAPI; AppliChem GmbH, Darmstadt, Germany). Briefly, 20 μl of a 100-μg ml^-1^ DAPI stock solution were added to sample and carefully vortexed (300 rpm; 2 min). Following 40 min incubation at 22°C, a portion of the coupon was covered with a glass cover slip and examined with the same fluorescence microscope described above. For the examination of the coupons, a 335–390 nm excitation filter as well as a 420–470 nm barrier filter was used. The maximum biofilm coverage was measured from the z-stack showing the highest level of coverage and sharpness of each of the 22 microscopic fields selected at random (63x/1.25 oil immersion objective). Results are described as percentage of coupons surface covered with cells and/or cell aggregates stained with DAPI. Images were taken and processed using the software ZEN Blue 2.5 (Carl Zeiss, Jena, Germany). In the supporting information, examples of microscopic observations show different degrees of microbial coverage on coupons (S3 Fig).

### Statistical analysis

Resulting bacterial (cfu) and cell counts of the two conducted trials were converted to log_10_-values for ease of interpretation and conduction of statistical analysis. Due to the initial differences on counts recorded in water samples between treated and non-treated lines, the effect of treatment alone was assessed by calculating the reduction (log_10_). Reduction was calculated for consecutive sampling days as well as for the complete experiments duration (28 days). Statistical calculations were performed using SAS software version 9.4 for windows (SAS Institute Inc., Cary, NC, USA). The results of the statistical tests were considered significant for *p*<0.05. Initially only for non-treated lines, cfus, cell counts as well as bacterial biofilm coverage were analyzed with a general linear model for mixture distributions (PROC GLIMMIX) considering the potential influencing factors: trial, drinking system type, and sampling point position. Hereby, the days of measurement were established as a repeated factor. In those cases, for which significant differences (*p*<0.05) were observed for the factor “trial”, further statistical calculations (PROC GLIMMIX) were performed to assess the degree of influence on observations of any uncontrollable factor present in every trial for each drinking system type. Finally, data arrays of water and biofilm samples were compared at each day between treated and non-treated lines for each drinking system type separately, using the Wilcoxon signed-rank test.

## Results

### Temperature and pH

The mean temperature of the water sampled at the nipples of controls and treated water systems ranged from 22°C to 24°C throughout the experiments. Mean temperatures of both control and treated lines of both systems were virtually equal at every sampling day (differences < 0.5°C). Similarly, mean pH values of control and treated water lines differed only minimally over the 28 days. Mean values of water pH as well as water temperature for every system at each sampling day are included in the supporting information (S2 and S3 Tables).

### Effect of EMFs on culturable bacterial counts and cell counts of water samples

**Fig 2:**
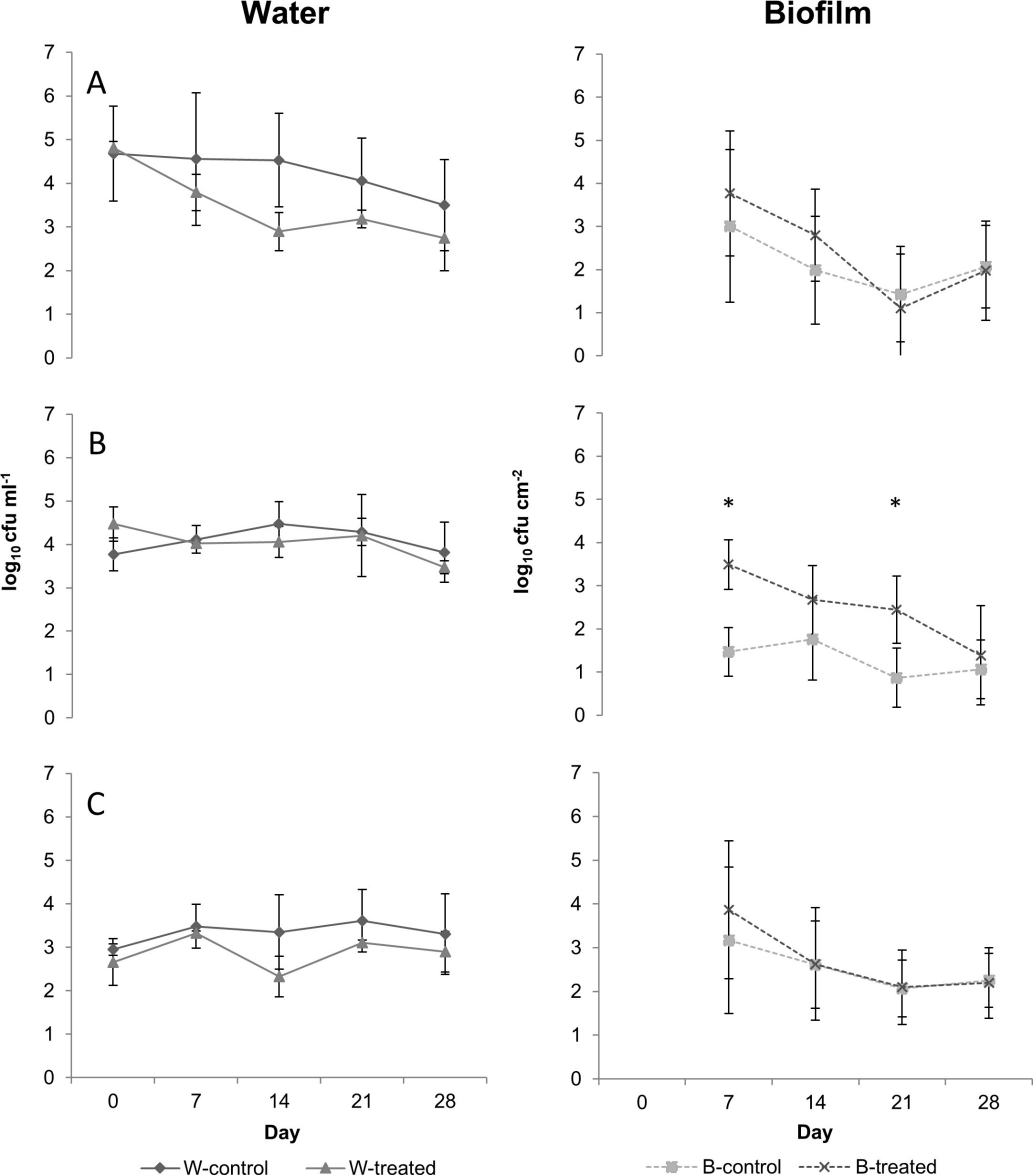
**Counts of culturable bacteria (log**_**10**_ **cfu) for heterotrophs growing at 22°C (A), heterotrophs growing at 36°C (B), and pseudomonads (C) in circulating systems.** W-control and W-treated as well as B-control and B-treated represent values obtained from water and biofilm samples, respectively. * Counts significantly different between treated and control at sampling day (Wilcoxon signed-rank test; *p*<0.05). Each value represents the mean of data arrays obtained from two independent experiments. Bars represent SD (water samples *n* = 2; biofilms samples *n* = 6).

**Fig 3:**
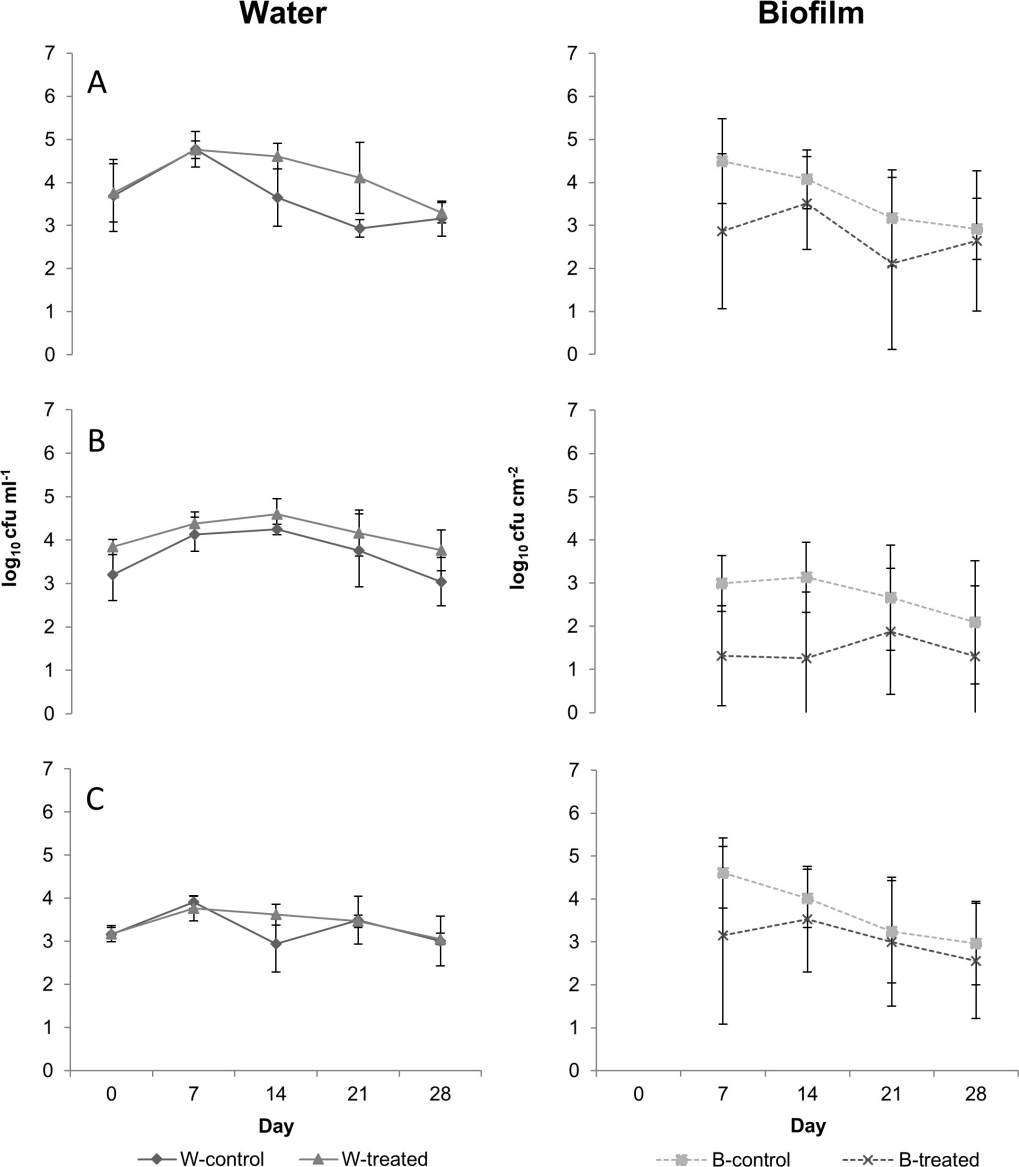
**Counts of culturable bacteria (log**_**10**_ **cfu) for heterotrophs growing at 22°C (A), heterotrophs growing at 36°C (B), and pseudomonads (C) in non-circulating systems.** W-control and W-treated as well as B-control and B-treated represent values obtained from water and biofilm samples, respectively. Each value represents the mean of data arrays obtained from two independent experiments. Bars represent SD (water samples *n* = 2; biofilms samples *n* = 6).

Initial statistical analysis revealed that colony counts of water differed significantly neither between trials nor between the drinking systems studied (Table 1). As shown in Figs 2 and 3, heterotrophs growing at 36°C and pseudomonads obtained from water samples of controls and from both treated circulating and treated non-circulating water showed similar trends with only small differences in the development patterns (fluctuations of ≤ 1-fold) over 28 days of the experiment (Figs 2 and 3). Only HPC-22 showed greater deviations between treated and control water lines of both systems, however without statistical significance (*p*>0.05). Briefly, planktonic HPC-22 in control circulating system decreased constantly throughout the experiments from 4.68 to 3.50 log_10_ cfu ml^-1^, while the decrease in treated line accounted 2-folds (Fig 2). Contrary to circulating lines, HPC-22 of non-circulating both treated and control systems showed virtually equal values at the beginning and to the end of the experiment, with obvious but statistically not significant differences at days 14 and 21 (Fig 3).

**Table 1:** *P*- values of general linear model for mixture distributions (PROC GLIMMIX) for water and biofilm samples.

		Trial	Drinking system type	Sampling point position
Water	HPC-22	0.2500	0.2864	-
	HPC-36	0.1652	0.7505	-
	Pseudomonads	0.3387	0.7855	-
	Bell counts	0.1056	0.0135	-
Biofilm	HPC-22	0.1012	0.0113	0.8107
	HPC-36	0.6337	<0.0001	0.1624
	Pseudomonads	0.1983	0.0122	0.8081
	Bacterial coverage	0.0061	0.0006	0.9410

Statistical significance with *p*<0.05

For the microscopic examination of water samples, statistical analysis showed with *p* = 0.0135 significance in the differences accounted between untreated lines of both drinking systems (Table 1). However, with regard to LF-EMF treatment for each drinking system separately, epifluorescence showed that the evolution of mean counts of cells suspended in water did not differ between control and treated lines. Briefly, mean counts of all systems ranged between 5.91 ± 0.03 log_10_ cells ml^-1^ at the beginning and 6.29 ± 0.05 log_10_ cells ml^-1^ at the end of the experiments, although with higher but not significant alterations in the means of days 7 and 21 (± 0.10 log_10_ cells ml^-1^; *p*> 0.05) (Table 2). Furthermore, ratio of cells stained with PI and those stained with SYTO 9 remained under 5% without any significant differences between treated and non-treated lines of both systems (Table 3).

**Table 2:** Total cell concentration (log_10_ cells ml^-1^) in water of control and LF-EMF treated drinking systems.

Day	Circulating		Non-circulating	
	Control	Treated	Control	Treated
0	5.87 ± 0.00	5.92 ± 0.37	5.91 ± 0.05	5.92 ± 0.08
7	6.16 ± 0.11	5.94 ± 0.19	6.01 ± 0.11	6.10 ± 0.22
14	6.34 ± 0.14	6.28 ± 0.08	6.23 ± 0.30	6.27 ± 0.18
21	6.27 ± 0.34	6.00 ± 0.24	6.08 ± 0.33	6.10 ± 0.16
28	6.34 ± 0.01	6.31 ± 0.15	6.23 ± 0.06	6.25 ± 0.08

Each value represents the mean of data arrays obtained from two independent experiments ± SD (*n* = 2).

**Table 3:** Ratio of PI-stained cells and total cells (%) in water of control and LF-EMF treated drinking systems.

Day	Circulating		Non-circulating	
	Control	Treated	Control	Treated
0	2.4 ± 2.1	2.9 ± 0.8	4.4 ± 1.2	2.5 ± 1.9
7	3.5 ± 1.1	2.6 ± 3.3	3.5 ± 2.5	3.2 ± 2.3
14	3.2 ± 0.4	3.9 ± 0.1	2.9 ± 1.5	2.4 ± 2.0
21	1.7 ± 0.5	2.4 ± 0.6	4.4 ± 0.3	5.2 ± 2.0
28	1.9 ± 0.1	3.4 ± 1.3	3.2 ± 1.3	4.4 ± 1.1

Each value represents the mean of data arrays obtained from two independent experiments ± SD (*n* = 2).

### Effect of EMFs on culturable bacterial counts of biofilm samples and biofilm coverage on PVC-coupons

As determined by initial statistical analysis, colony counts on PVC-coupons of control circulating and non-circulating systems developed differently during the experiments (Table 1). Generally, mean counts of biofilms of control circulating line remained lower than those of the non-circulating water line for HPC-22, HPC-36 and number of pseudomonads (Figs 2 and 3). Regarding LF-EMF treatment, exposition did not influence the development patterns of HPC-22 and the counts of pseudomonads in biofilms during the experiments (Figs 2 and 3). In contrast, HPC-36 of treated lines were considerable different to the counts obtained from controls of both circulating and non-circulating systems. HPC-36 counts of control biofilms of non-circulating system were considerably higher than the counts of treated line, especially in the first 2 weeks of experiments, however without statistical significance (*p* = 0.07 for day 7 and *p* = 0.13 for day 14, respectively) (Fig 3). Otherwise, in circulating systems, HPCs-36 in biofilm were higher in the treated line compared to the control (Fig 2). Differences between counts were clearer over 21 days of experiments with statistical significances at day 7 (*p* = 0.0051) and day 21 (*p* = 0.0124). To the end of the experiments however, HPC-36 of both control and treated circulating systems accounted means of 1.07 log_10_ cfu cm^-2^ and 1.39 log_10_ cfu cm^-2^, respectively (Fig 2).

**Fig 4:**
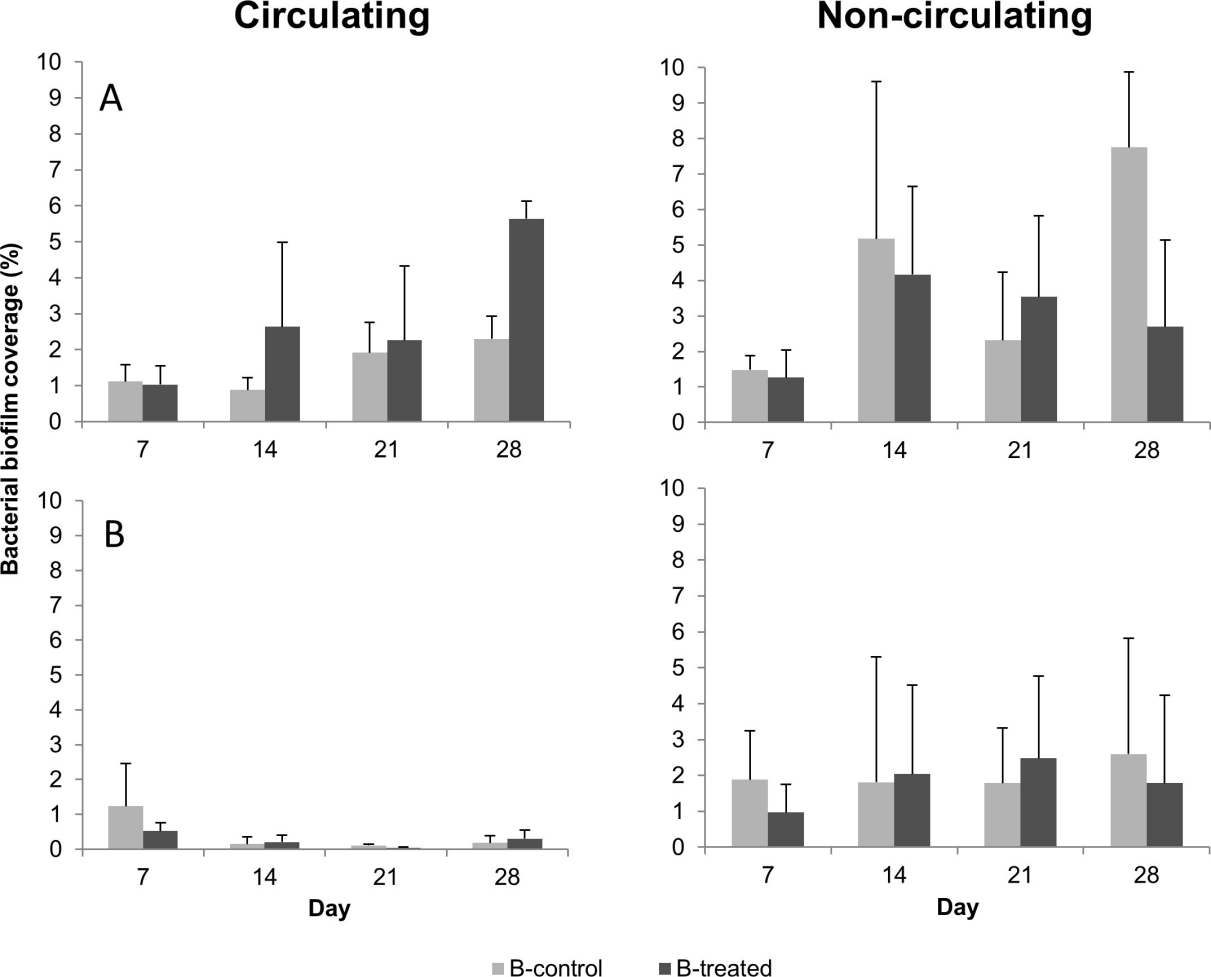
**Percentage coverage of bacterial biofilm coverage on PVC-coupons in circulating and non-circulating drinking systems in the first (A) and second trial (B).** Each value represents the mean of data arrays obtained from each of the two independent experiments. Bars represent SD (*n* = 6).

Regarding microscopic results, initial statistical analysis showed that biofilm development in controls of both system types differed significantly between both trials (Table 1). Generally, microscopic analyses indicated a more effectively spread of biofilm in the first trial compared to the second trial (Fig 4). Subsequent statistical analysis (general linear model) revealed highly significant variations between trials for control lines of the circulating system (*p* = 0.0025). For non-circulating systems however, differences between both trials merely approached the borderline of significance (*p* = 0.0507). Hence, results of bacterial coverage with regard to LF-EMF treatment were analyzed separately per trial. Development pattern of control and treated biofilms over 28 days experiment is shown for every trial in Fig 4.

In non-circulating systems, biofilms of both control and treated lines covered after initial colonization similar areas during the first 21 days of the first trial. At 28 day however, biofilm of control line covered 7.8% of the total surface of coupon, whilst biofilm of treated line spread over 2.7% (*p* = 0.0809) (Fig 4). In the second trial, the spread pattern of biofilms in control and treated non-circulating systems were similar over the complete duration of the experiment. Similarly, and regardless of the general differences in development patterns between trials described above, percentage of coverage was similar in both treated and untreated circulating lines, with one exception: at day 28 of the first trial, biofilm of the exposed line covered 3% more area than its counterpart of the control line (p = 0.3827) (Fig 4).

## Discussion

Water offered to farm animals should comply with the highest levels of quality in order to guarantee the highest quality and safety of the products from those animals. Biofilm formation in water lines of drinking systems impairs the general microbiological quality of drinking water and may present a risk to animal health [4]. Since the use of LF-EMF has been shown to be a promising alternative to traditional chemical cleaning of water systems of animal productions [14], the effects of this treatment on bacterial water bacterial microbiota, including the culturable cells on blood-agar base and those stained with DNA-binding fluorophores, in 20 m-long models of drinking systems used in poultry industry was tested for 28 days. Through the presented approach, this study aimed to obtain basic information on the usability of this kind of technologies in water supply lines of poultry premises. For the determination of culturable bacteria, the non-selective blood-agar base was chosen over others typically recommended for water samples (e.g. R2A-agar). The long incubation periods and the especial components of the media recommended for the analysis of water samples may favor not only the growth of water microorganisms [30] but also that of damaged or stressed cells [31]. Hence, this characteristics may have had distorted the perception on bacterial culturability of bacteria affected by LF-EMF treatment. By this experimental approach, potential influencing factors, such as animal drinking habits or the retrograde entrance of bacteria in water via nipples, were excluded, which was important for this first evaluation step. To the best of our knowledge, this is the first published data about the effects of LF-EMF on waterborne bacteria in models of commercially available drinking systems used in poultry holdings.

As determined by plate counts, LF-EMF treatment may have an effect on bacterial microbiota since treatment resulted on considerable variations in colony counts of bacteria suspended in water or adhered to PVC-surfaces. Although with high standard deviations for some data arrays, counts of culturable bacteria seemed to be differently influenced by exposition. The results of the present study are in agreement with observations of Mercier et al. [17], showing changes in the planktonic and sessile bacterial community structure of water microbiota during exposition to LF-EMF (1–5 mT, 1–10 kHz) by terminal restriction fragment length polymorphism (t-RLPF). As sensitivity to electromagnetic fields differs between bacterial strains [32], treatment may disfavor the development of certain bacteria, which results in modifications of general bacterial composition of treated water [17]. Interestingly, in the present study, the counts of culturable bacteria of each affected community, especially of heterotrophs growing at 22°C and 36°C for water and biofilm samples, respectively, was either reduced or increased by treatment. Latter differed between the drinking system types studied. The resulting inhibition of cell growth observed in non-circulating lines may be related to reduction of Adenosine triphosphate (ATP), protein and polysaccharide production during exposition to LF-EMFs, as reported by others using similar frequencies (1–100 kHz) and amplitudes of 1–5 mT [14, 17]. In contrast, exposure of water in circulation led to an increase of colony counts of heterotrophs, especially in biofilms. This was not expected, since water circulated continuously through the device having periodically direct contact with the electromagnetic signal source. When comparing both drinking systems, the pump as well as the special architecture of circulating lines were the only relevant components posing an additional factor to the experiments. Therefore, we hypothesize (1) that the direct mechanical action of the pump and/or (2) the resulting change of water flow dynamics during circulation through water lines may have affected some characteristics of electromagnetic waves during propagation [33] and may have consequently induced a different bacterial response. Other studies have shown that the direct application of weaker electromagnetic fields (≤ 100 Hz; ≤ 1 mT) on bacterial cultures may have not only reductive [24, 25, 32], but also neutral [21, 34], or even stimulatory effects on bacterial cell growth [19, 20]. Interestingly, Piyadasa et al. [35] reported that both stimulatory and inhibitory effects could be observed on the culturability of *Escherichia coli* and *Pseudomonas fluorescence* by 7 h exposure to pulsed electromagnetic fields under copiotrophic conditions. Latter authors showed that outcomes were influenced by water flow conditions (static vs. low flow vs. high flow) and differed between the two commercial devices used [35]. Therefore, we assume that the bacterial microbiota of circulatory systems may have been exposed to a different treatment regime, which induced temporarily an increase of colony counts, especially of heterotrophic bacteria growing at 36°C.

Additionally to culturable counts, the viability of planktonic microorganisms seemed not to be affected by LF-EMF treatment, as neither total cell counts nor live/dead ratio differed between treated and controls. Latter regardless of the drinking system studied. Furthermore, no inhibitory effects of LF-EMF treatment on biofilm formation were confirmed over 28 days by fluorescence microscopy. Microscopic results on planktonic cells are in agreement with previous studies showing no biocide effects of EMF in pure cultures of *E*. *coli* [19, 20, 34] or in complex microbiota of river water or sludge [14, 17, 26]. However, decreases in viability of cells in monoculture biofilms of *Helicobacter pylori* [21], as well as affection of cell division mechanisms and molecular DNA structure of planktonic laboratory-grown *Salmonella typhi* have also been reported [25]. Interestingly, the substantial fluctuations observed by colony counts in the present study were not in accordance with microscopic observations, as the concentration of stained cells in water samples as well as the bacterial colonization of coupons remained constant throughout the experiments. Furthermore, as exposure continued, colony counts of treated and control drinking water systems converged to the end of experiments (Figs 2 and 3). Previous studies reported that LF-EMF treatment of different frequencies applied directly on cultures may affect cell metabolism, as exposure caused variations in different parameters used to evaluate bacterial activity [19, 20, 21, 24, 34, 36]. Additionally, adaptive responses have been reported in *E*. *coli* and *P*. *aeruginosa* exposed directly to extremely low-frequency electromagnetic fields (2.0 mT; 50 Hz) under laboratory conditions after 24 h [37] as well as in bacterial microbiota of water reactors [26]. In the case of the water reactors, lower bacterial counts were determined in forming biofilms of treated water reactors compared to those from controls during 30 days exposure. As the treatment continued, differences disappeared and remained absent over the following two months of the experiments [26]. In accordance with previous studies, our data suggest that low-frequency electromagnetic waves of the applied regime may induce an initial response of microorganisms, which influences cell metabolism, division and results in alterations of culturable counts in drinking systems. As exposure continues however, bacteria may adapt to new conditions and return to the initial activity.

Regarding cell adhesion, in the present study, there was no indication that LF-EMF treatment had an effect on attachment properties of cells into water, as biofilm developed similarly and the number of cells suspended in water remain stable in treated and control lines. In contrast, LF-EMF exposure has been reported to cause modifications of membrane structures affecting cell adhesion and aggregation, resulting consequently in an increased number of planktonic cells and a decrease of biofilm formation during exposition [14, 17, 19]. It is important to note, that 28-days experiment may not be adequate to completely exclude possible effects of LF-EMFs on cell attachment and consequently on biofilm formation, since statistically significant variations may be measurable only after longer periods of exposition [14, 17]. Additionally, despite comparability of conditions in terms of water exposure, differences between trials indicate that uncontrollable factors, present by this type of approach (e.g. water properties within trials or corrosion), may have an influence on the general outcome of the experiments [26]. Moreover, bacteria represent just a part of the biofilm matrix and the determination of their numbers in biofilm alone may not allow the estimation of mineral scaling or polysaccharide content of biofilms, which seems to be affected by LF-EMF exposure [14, 16].

Regarding structural differences between drinking systems without LF-EMF treatment, continual circulation of water through water lines (0.7 l min^-1^) affected accumulation of biofilm on coupons surface. Latter observations were determined by culture methods as well as through fluorescence microscopic examinations and confirmed with statistical analysis (Table 1). Hydrodynamic stress has been experimentally shown to reduce growth rate [38], and influence EPS-to-cell ratios of forming biofilms [39], as well as to have a negative impact on cohesion properties, bacterial density and general organization of established biofilms [40]. The present study confirms the relationship between hydraulics and biofilm development also in drinking water distribution networks used in animal husbandry.

## Conclusions

In conclusion, data of this study indicates that LF-EMF exposure of water in drinking systems resulted partially in temporal alterations of bacteria culturability, although without biocide effects. However, in a period of 28 days, LF-EMF treatment of water did not have considerable effects on colony counts of planktonic or biofilm microorganisms in models of drinking systems used conventionally in poultry premises. Since parameters varied in control drinking systems between separate trials, additional factors may also have influenced bacterial microbiota during the experiments, alone or in combination with LF-EMF treatment. Nevertheless, this study included merely two different types of drinking systems used conventionally in poultry industry. Since results differed between drinking system types and drinking system architectures vary in and within animal holdings, it is not possible to exclude clearer effects in drinking systems of other animal productions. Altogether, results suggest that LF-EMF treatment alone may not replace conventional sanitation methods and hygiene management of water systems at present conventionally used in poultry premises. In accordance to Piyadasa et al. [41], it is estimated that further scientific validation is needed as well for the application of EMF technologies in animal husbandry in the future. Based on the presented data, cooperative development of devices technology and drinking system architectures may be required. In addition to bacterial culture methods and fluorescence microscopy, future evaluations of the applicability of LF-EMF technologies in practice may include additional methods for describing both the bacterial community exposed as well as its metabolic activity and biofilm matrix composition. Furthermore, interactions between hydraulic pattern and biofilm formation may be taken into account when designing the architectural configuration of water distribution networks in animal production systems.

## Supporting information

S1 Fig Images of models of drinking systems. (A) Room 1: water supply, control and treatment unit for non-circulating drinking systems. (B) Room 2: Models of drinking water systems (left: treated; right: control).
(TIF)

S2 Fig Example microscopic images of cell and cell aggregates of water samples on polycarbonate filters. (A) and (B) Microscopic fields predominantly covered with single cells. (C) and (D) Presence of single cells and cell aggregates.
(TIF)

S3 Fig Example microscopic images of cell and cell aggregates on PVC-coupons. (A) Microscopic field predominantly covered with single cells. (B), (C) and (D) Different development stages of bacterial biofilm with presence of single cells and cell aggregates.
(TIF)

S1 Table Inlet water characteristics. (DOCX)

S2 Table Mean pH values of water at each sampling day during experiments. Each value represents the mean of data arrays obtained from two independent experiments ± SD (*n* = 6).
(DOCX)

S3 Table Mean temperature of water at each sampling day during experiments. Each value represents the mean of data arrays obtained from two independent experiments ± SD (*n* = 6).
(DOCX)
