# Supplementary figures and images for: Low-frequency electromagnetic fields as an alternative to sanitize water of drinking systems in poultry production?
Source: PLoS One. 2019 Jul 25;14(7):e0220302. doi: 10.1371/journal.pone.0220302 (PMC6657887; doi:10.1371/journal.pone.0220302)

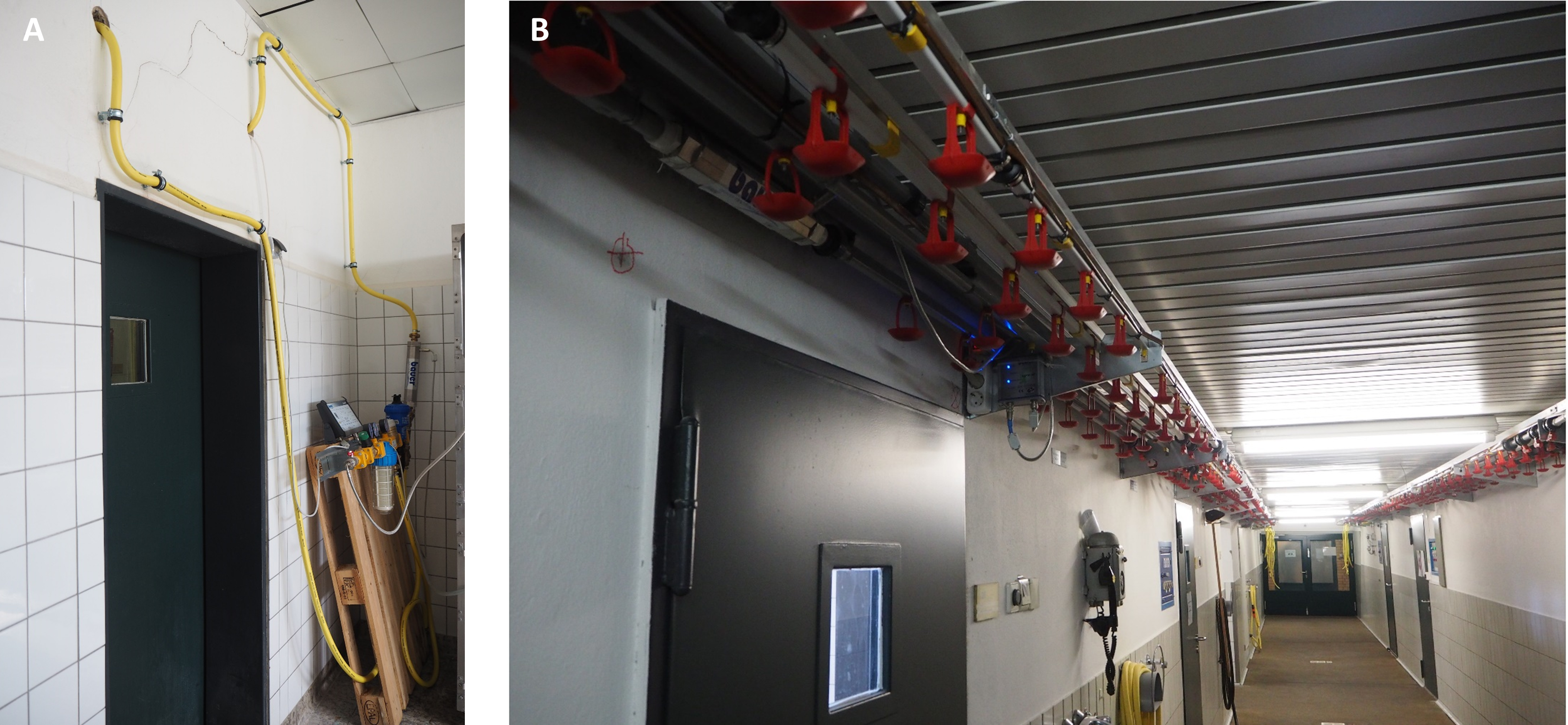

Supplement: S1 Fig — (A) Room 1: water supply, control and treatment unit for non-circulating drinking systems. (B) Room 2: Models of drinking water systems (left: treated; right: control). (TIF) [file pone.0220302.s001.tif]

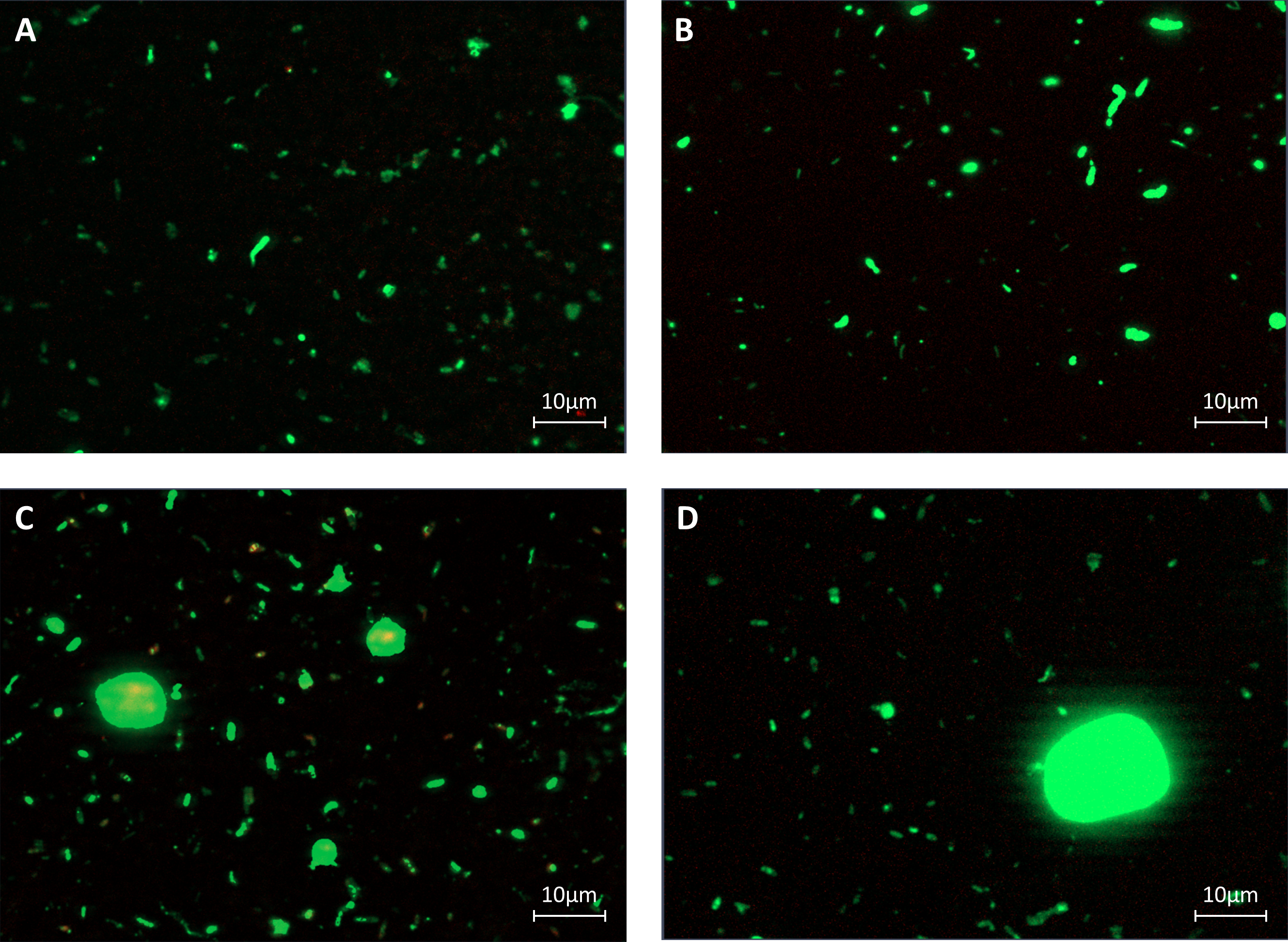

Supplement: S2 Fig — (A) and (B) Microscopic fields predominantly covered with single cells. (C) and (D) Presence of single cells and cell aggregates. (TIF) [file pone.0220302.s002.tif]

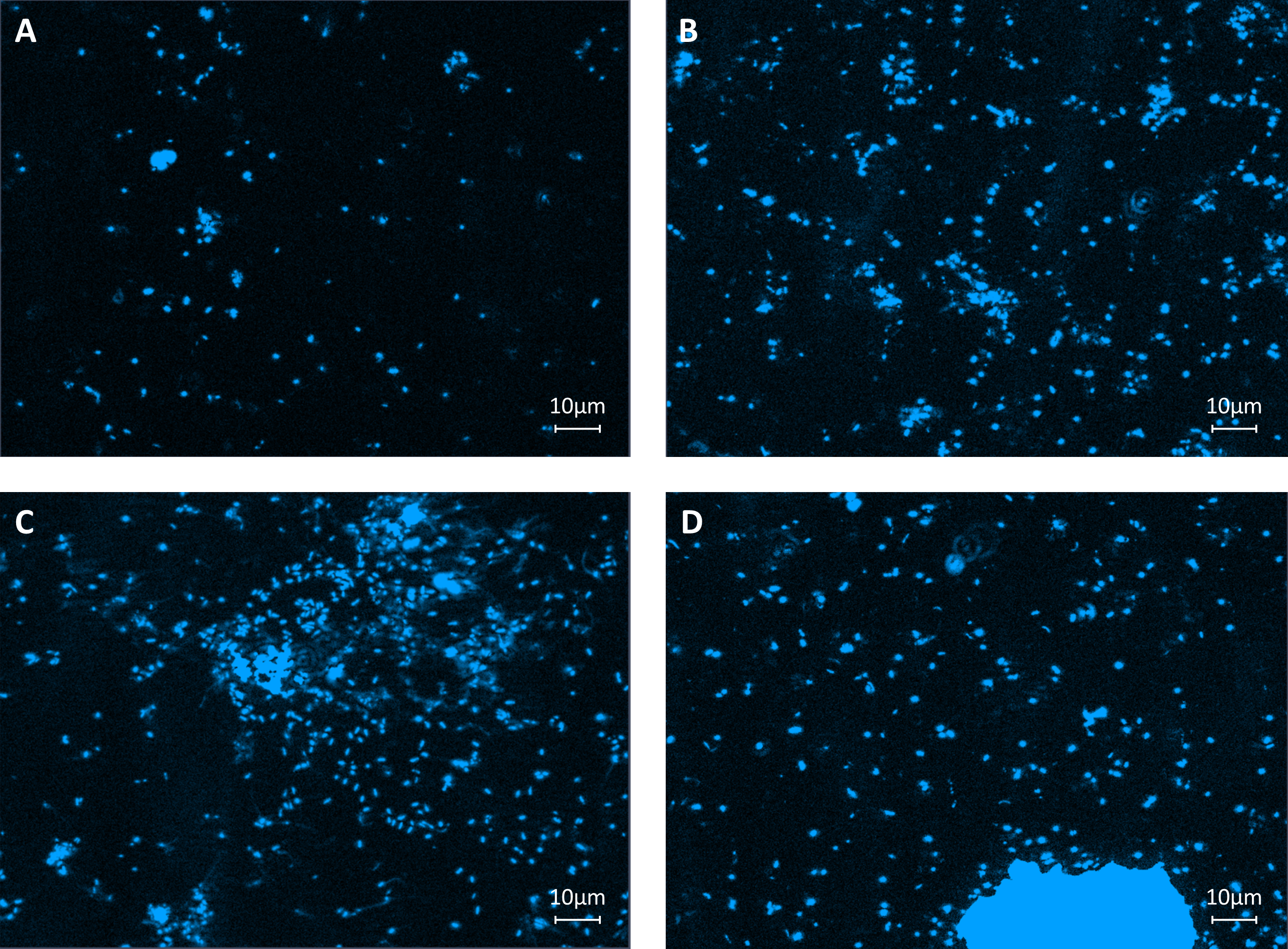

Supplement: S3 Fig — (A) Microscopic field predominantly covered with single cells. (B), (C) and (D) Different development stages of bacterial biofilm with presence of single cells and cell aggregates. (TIF) [file pone.0220302.s003.tif]
