# Supplementary material for: Low-frequency electromagnetic fields as an alternative to sanitize water of drinking systems in poultry production?
Source: PLoS One. 2019 Jul 25;14(7):e0220302. doi: 10.1371/journal.pone.0220302 (PMC6657887; doi:10.1371/journal.pone.0220302)
Supplement: S1 Table — (DOCX) [file pone.0220302.s004.docx]

| Parameter | Unit | Value |
| --- | --- | --- |
| Bacterial counts | cfu 100 ml^-1^ | 4.5 |
| pH |  | 7.66 |
| Temperature | °C | 20.7 |
| Conductivity | µS cm^-1^ | 568 |
| Dissolved oxigen | mg l^-1^ | 8.66 |
| Turbidity | FNU | <1 |
| NH_4_^+^ | mg l^-1^ | <0.04 |
| PO_4_^3-^ | mg l^-1^ | 0.35 |
| Na | mg l^-1^ | 21.8 |
| K | mg l^-1^ | 3.27 |
| Ca | mg l^-1^ | 68.4 |
| Mg | mg l^-1^ | 4.54 |
| Fe | mg l^-1^ | <0.02 |
| Mn | mg l^-1^ | <0.02 |
| Cl^-^ | mg l^-1^ | 43.0 |
| NO_2_^−^ | mg l^-1^ | <0.1 |
| NO_3_^−^ | mg l^-1^ | 2.06 |
| SO_4_^2−^ | mg l^-1^ | 90.9 |
| TOC | mg l^-1^ | 4.87 |
