# Supplementary material for: Low-frequency electromagnetic fields as an alternative to sanitize water of drinking systems in poultry production?
Source: PLoS One. 2019 Jul 25;14(7):e0220302. doi: 10.1371/journal.pone.0220302 (PMC6657887; doi:10.1371/journal.pone.0220302)
Supplement: S2 Table — Each value represents the mean of data arrays obtained from two independent experiments ± SD (n = 6). (DOCX) [file pone.0220302.s005.docx]

| Day | Circulating | | Non-circulating | |
| --- | --- | --- | --- | --- |
|  | Control | Treated | Control | Treated |
| 0 | 7.52 ± 0.06 | 7.64 ± 0.04 | 7.81 ± 0.74 | 7.76 ± 0.54 |
| 7 | 7.09 ± 0.52 | 7.16 ± 0.50 | 7.62 ± 0.85 | 7.46 ± 0.57 |
| 14 | 6.85 ± 0.09 | 6.98 ± 0.13 | 7.44 ± 1.00 | 7.00 ± 0.16 |
| 21 | 6.92 ± 0.16 | 7.01 ± 0.11 | 7.36 ± 0.86 | 7.07 ± 0.15 |
| 28 | 6.80 ± 0.04 | 6.82 ± 0.08 | 6.92 ± 0.15 | 6.84 ± 0.12 |
