# Supplementary material for: Low-frequency electromagnetic fields as an alternative to sanitize water of drinking systems in poultry production?
Source: PLoS One. 2019 Jul 25;14(7):e0220302. doi: 10.1371/journal.pone.0220302 (PMC6657887; doi:10.1371/journal.pone.0220302)
Supplement: S3 Table — Each value represents the mean of data arrays obtained from two independent experiments ± SD (n = 6). (DOCX) [file pone.0220302.s006.docx]

| Day | Circulating | | Non-circulating | |
| --- | --- | --- | --- | --- |
|  | Control | Treated | Control | Treated |
| 0 | 23.6 ± 0.9 | 23.8 ± 1.0 | 23.4 ± 0.9 | 23.4 ± 0.8 |
| 7 | 23.2 ± 0.5 | 23.2 ± 0.4 | 23.0 ± 0.5 | 23.0 ± 0.4 |
| 14 | 21.9 ± 0.9 | 22.6 ± 1.1 | 22.1 ± 0.9 | 21.8 ± 0.9 |
| 21 | 22.5 ± 1.6 | 22.9 ± 1.2 | 22.5 ± 1.1 | 22.5 ± 1.4 |
| 28 | 22.5 ± 1.4 | 22.7 ± 1.2 | 22.3 ± 1.0 | 22.2 ± 1.1 |
